# Frequency and risk factors for venous thromboembolism after gastroenterological surgery based on the Japanese National Clinical Database (516 217 cases)

**DOI:** 10.1002/ags3.12275

**Published:** 2019-07-22

**Authors:** Taishi Hata, Masataka Ikeda, Hiroaki Miyata, Masatoshi Nomura, Mitsukazu Gotoh, Masato Sakon, Kouji Yamamoto, Go Wakabayashi, Yasuyuki Seto, Masaki Mori, Yuichiro Doki

**Affiliations:** ^1^ Department of Gastroenterological Surgery Graduate School of Medicine Osaka University Suita Osaka Japan; ^2^ Department of Surgery Hyogo College of Medicine Nishinomiya Hyogo Japan; ^3^ Department of Health Policy and Management School of Medicine Keio University Shinjuku‐ku Tokyo Japan; ^4^ The Japanese Society of Gastroenterological Surgery Minato-ku Tokyo Japan; ^5^ Osaka International Cancer Institute Osaka Osaka Japan; ^6^ Department of Biostatistics School of Medicine Yokohama City University Yokohama Kanagawa Japan; ^7^ Database Committee The Japanese Society of Gastroenterological Surgery Minato‐ku Tokyo Japan; ^8^ Department of Surgery and Science Graduate School of Medical Sciences Kyushu University Higashi‐ku Fukuoka Japan

**Keywords:** gastroenterological surgery, National Clinical Database, pulmonary embolism, venous thromboembolism

## Abstract

**Aim:**

To investigate the frequency and risk factors of perioperative, symptomatic venous thromboembolism (VTE) after gastroenterological surgery.

**Methods:**

We assessed the frequency of and risk factors for VTE after eight gastroenterological procedures (total 516 217 cases including, gastrectomy, total gastrectomy, hepatectomy, esophagectomy, right hemicolectomy, low anterior resection, pancreaticoduodenectomy, and acute pan‐peritonitis surgery) based on data from the National Clinical Database. Data collected between 2011 and 2013 (382 124 cases) were used as a test set, and data from 2014 (134 093 cases) were used as a validation set.

**Results:**

The frequency of deep vein thrombosis (DVT) was 0.3% (382 124 cases), and the incidence of pulmonary embolism (PE) was 0.2% (382 124 cases) ranging from 0.1% to 0.7% for DVT and from 0.1% and 0.3% for PE among eight surgeries, respectively. Analyses using pre‐and intra‐operative factors identified the top three risk factors for VTE as esophagectomy, pancreaticoduodenectomy, and hepatectomy. Using pre‐, intra‐, and postoperative factors, the second through fourth risk factors were sepsis, prolonged ventilation >48 hours and readmission within 30 days. The highest risks factor for PE using pre‐, intra‐, and postoperative factors were any cardiac events. Unplanned intubation was the fourth risk factor.

**Conclusion:**

The risk for DVT and PE differed for each surgical procedure. VTE and PE risk factors changed after considering postoperative factors. It may be necessary to reconsider the prophylaxis depending on whether the complication occurs after surgery, particularly breathing and cardiac complications.

## 1 INTRODUCTION

Venous thromboembolism (VTE) is a well‐recognized, common surgical complication in European countries. The incidence of fatal VTE ranges from 0.1% to 0.8%.^1^ However, a recent survey indicated that the age‐adjusted mortality rate from VTE markedly increases.^2^ According to a recent study in Japan, VTE occurred in 24.3% of patients undergoing abdominal surgery, including asymptomatic cases.^3^ This result shows that the incidence of VTE in Japan is not inferior in comparison with Western countries.

The risk of VTE differs across various patient and disease factors and types of operation. The risk of VTE is highest during the initial period after the diagnosis of malignancy.^4^ VTE, including asymptomatic cases, occurs in up to 20% of cancer patients and is one of the leading causes of death in patients with cancer.^5^, ^6^ VTE is a potentially fatal but preventable complication after major gastroenterological surgery for cancer.^7^ However, the incidence of symptomatic VTE is very low during the operation, requiring a large sample size to detect risk factors in detail.

The National Surgical Quality Improvement Program (NSQIP) of the American College of Surgeons provides practical and real‐time clinical data about surgical risks to surgeons.^8^, ^9^ On the other hand, in Japan the National Clinical Database (NCD), a nationwide project that is linked to the surgical board certification, started patient registration in January 2011.^10^, ^11^ This prospective, multicenter clinical registry was created to provide feedback on risk‐adjusted outcomes to hospitals and surgeons for quality improvement purposes. This system covers more than 90% of general surgeries performed in Japan, with 9 690 000 patients from more than 5100 hospitals registered each year.

The NCD collects reliable and validated data, including demographics, laboratory results, comorbidities, and postoperative outcomes for patients undergoing a range of surgeries in Japan. In this study, using the NCD system, we identified the risk factors for perioperative VTE for patients undergoing gastroenterological surgeries.

## 2 METHODS

### 2.1 Patient selection (data collection)

Japan's NCD is a nationwide project in cooperation with the certification board of the Japan Surgical Society. From January 2011, data for more than 9 690 000 patients undergoing surgery were collected from more than 5100 hospitals registered each year. Information about operations performed nationwide was registered in the NCD by data management departments from the participating institutions (http://www.ncd.or.jp/). The data were evaluated annually using a web‐based data management system to assure data traceability.^12^ This system also validates data consistency by inspecting randomly chosen institutions.

The NCD is continuously updated by data management departments from participating institutions and is evaluated annually using an internet‐based data management system to ensure data quality. Current laws, ordinances, and guidelines regarding the confidentiality of data are observed. Patients agree to have their data included in research projects by using presumed consent with opt‐out through the Web page and/or a notice at each hospital.

In this study, we used eight gastroenterological procedures (gastrectomy, total gastrectomy, hepatectomy, esophagectomy, right hemicolectomy, low anterior resection, pancreaticoduodenectomy, and acute pan‐peritonitis surgery) data. Because these data were determined to represent the performance of surgery in each specialty, the input of detailed items such as laboratory data and operative morbidities was requested.

Within these procedures, there were 382 124 cases between 2011 and 2013 and 134 093 cases in 2014. We used data between 2011 and 2013 as a test set to develop the prediction model and data from 2014 as a validation set.

### 2.2 Endpoint

The primary objective was to investigate the frequency of VTE and pulmonary embolism (PE) in actual clinical practice from data in the NCD. A secondary objective was to identify the risk factors for perioperative VTE and PE.

### 2.3 Prediction model development

We developed two different risk models for predicting postoperative VTE. One used pre‐ and intra‐operative factors. In the other model, postoperative factors were added. PE was analyzed separately using a similar approach.

Univariate analysis was done using Fisher's exact test and the two‐tailed *t* test. In the development of the dataset, we built multivariable logistic regression models using a forward step‐wise selection of predictors with a *P *=* *0.05 for entry and *P *=* *0.10 for exit.

Basically, in cross‐tabulation with the responding outcome, variables that were <0.2 and variables that seem to be clinically significant even if some *P* > 0.2 were added.

Even if these were a similar event, we used the combination of categories that were logically consistent and clinically relevant from a clinical point of view.

We assessed the models’ performances by applying the predictor to the validation dataset and evaluating its ability to discriminate between the presence and absence of VTE or PE using the C‐index, which reflects the area under the receiver operating characteristic (ROC) curve for VTE or PE. A ROC curve is a plot of a test's true‐positive rate (sensitivity) versus its false‐positive rate (1 ‐ specificity). Each point on the ROC curve indicates a pair of false‐ and true‐positive rates that is achieved using a particular threshold to dichotomize the predicted probabilities. All statistical analyses were performed using SPSS (version 21; IBM Corp., Armonk, NY, USA). This study was approved by the institutional review board of Japan Surgical Society.

## 3 RESULTS

### 3.1 Incidence of DVT and PE

The incidence of deep vein thrombosis (DVT) and PE associated with the eight gastroenterological procedures are shown in Table 1.

**Table 1:** Incidence of VTE between 2011 and 2013 (NCD data, gastroenterological) (N = 382 124)

Operation	n	Pulmonary embolism (%)	n	Deep vein thrombosis (%)
Gastrectomy	93/101 481	0.10	123/101 481	0.12
Total gastrectomy	82/57 997	0.14	116/57 997	0.20
Hepatectomy	58/23 489	0.25	99/23 489	0.42
Esophagectomy	43/16 556	0.26	93/16 556	0.56
Right hemicolectomy	68/59 246	0.11	134/59 246	0.23
Low anterior resection	61/51 632	0.12	113/51 632	0.22
Pancreaticoduodenectomy	62/26 668	0.23	103/26 668	0.39
Acute pan‐peritonitis surgery	86/27 377	0.31	203/27 377	0.74
Total	553/382 124	0.14	984/382 124	0.26

Abbreviations: NCD, National Clinical Database; VTE, venous thromboembolism.

In the NCD data, PE was diagnosed by dynamic computed tomography (CT) scan, pulmonary arteriography, or by pulmonary blood flow scintigrams within 30 days after surgery. DVT was diagnosed by dynamic CT scan or duplex scan within 30 days after surgery. The incidence rate of DVT associated with the eight surgical procedures was between 0.1% and 0.7%.

The highest incidence rate (0.7%) was seen with acute pan‐peritonitis surgery followed by esophagectomy (0.6%). The incidence of PE was between 0.1% and 0.3%. The highest incidence (0.3%) was associated with esophagectomy and acute pan‐peritonitis surgery.

### 3.2 Patient characteristics

The patient population represented in the NCD had an average age of 68.6 years. The mean body mass index (BMI) of the population was 22.1 kg/m^2^. In the VTE‐positive group, BMI and creatinine levels were significantly higher and the estimated glomerular filtration rate was lower. Regarding the intra‐operative factors, the operation time was significantly longer and the loss of blood was greater in the PE‐positive group (Table 2).

**Table 2:** Patient characteristics (2011‐2013) (N = 382 124)

	Total	VTE (−)	VTE (+)	*P*	PE (−)	PE (+)	*P*
Mean age	68.6	68.6	70.7	0.003	68.6	70.3	0.198
Length of stay (d)	28.2	28.2	47.9	<0.0001	28.2	45.6	<0.0001
Length of ICU stay (d)	3.0	3.0	7.8	<0.0001	3.0	7.4	<0.0001
Height (cm)	159.7	159.7	158.6	0.243	159.7	159.1	0.367
Weight (kg)	56.7	56.7	57.6	0.504	56.7	58.8	0.547
BSA	1.58	1.58	1.58	0.762	1.58	1.60	0.97
BMI (kg/m^2^)	22.1	22.1	22.9	<0.0001	22.1	23.2	<0.0001
Creatinine (mg/dL)	0.89	0.89	1.00	<0.0001	0.89	0.98	0.001
eGFR	60.1	60.1	58.0	<0.0001	60.1	58.3	0.234
Anesthesia time (min)	339.6	339.4	404.6	<0.0001	339.5	403.8	<0.0001
Operation time (min)	277.3	277.1	336.4	<0.0001	277.2	338.2	<0.0001
Operation bleeding (mL)	432.9	431.8	760.6	<0.0001	432.5	731.5	<0.0001

Abbreviations: BMI, body mass index; BSA, body surface area; eGFR, estimated glomerular filtration rate; PE, pulmonary embolism; VTE, venous thromboembolism.

### 3.3 Risk profile associated with postoperative VTE and PE

There were no significant differences in the profiles of the variables between the data set and validation sets. All risk model data were derived from multivariable analysis.

### 3.4 Risk factors for VTE and PE

The final logistic regression models with odds ratios (ORs) were summarized. The 95% confidence intervals (CIs), statistical significance of each model and β‐coefficient intercept are presented.

The risk factor for VTE using the pre and intra‐operation factors included 23 factors (Table 3). The types of surgical procedure were among the top risk factors. The top three highest ORs were for esophagectomy (3.843), pancreaticoduodenectomy (2.349), and hepatectomy performed for >1 segment apart from the lateral segment (2.182). There was little relationship between sex (male, 0.759), disseminated cancer (1.321), or BMI >30 kg/m^2^ (1.442), which have previously been considered high‐risk factors for VTE.

**Table 3:** The risk for VTE associated with pre‐ and intra‐operative factors (N = 382 124)

	VTE risk preOP + OP			*P*
	β‐coefficient	Odds ratio	95% CI	
Age category^a^	0.184	1.202	1.111‐1.302	<0.001
Intrahepatic bile duct cancer	0.513	1.670	1.083‐2.573	0.02
ASA (grade3‐5)	0.316	1.371	1.186‐1.585	<0.001
No tumor	−0.19	0.827	0.655‐1.044	0.11
Hepatectomy performed for >1 segment apart from the lateral segment	0.780	2.182	1.782‐2.672	<0.001
Pancreaticoduodenectomy	0.854	2.349	1.973‐2.796	<0.001
Esophagectomy	1.346	3.843	3.166‐4.664	<0.001
Sex (male)	−0.276	0.759	0.679‐0.847	<0.001
Emergent surgery	0.656	1.927	1.523‐2.438	<0.001
ADL pre‐operation any assistance	0.210	1.234	1.039‐1.467	0.017
Pre‐operation ventilation	0.635	1.887	1.326‐2.687	<0.001
Hypertension any	0.146	1.157	1.033‐1.295	0.012
Recent cerebrovascular disease	0.613	1.846	1.162‐2.933	0.009
Disseminated cancer	0.278	1.321	1.034‐1.688	0.026
Chronic steroid use	0.534	1.706	1.245‐2.339	0.001
Weight loss over 10%	0.426	1.531	1.270‐1.847	<0.001
Bleeding disorder (any)	0.486	1.627	1.350‐1.960	<0.001
Systemic sepsis	0.771	2.162	1.717‐2.722	<0.001
BMI >25 kg/m^2^	0.585	1.794	1.572‐2.048	<0.001
BMI >30 kg/m^2^	0.366	1.442	1.095‐1.899	0.009
Platelet count >350 000/μL	0.212	1.236	1.046‐1.462	0.013
Serum albumin level <4 mg/dL	0.425	1.530	1.359‐1.723	<0.001
PT‐INR >0.9	0.340	1.405	1.047‐1.884	0.023

Abbreviations: ADL, activities of daily living; ASA, American Society of Anesthesiologists Physical Status Classification; BMI body mass index; CI, confidence interval; CRP, C‐reactive protein; OP, operation; preOP, preoperation; PT‐INR, prothrombin time/international normalized ratio; VTE, venous thromboembolism.

a The variables of age were categorized into four groups, which is less than 39, 40‐59, 60‐74, and more than 75 y. Therefore, this odds ratio indicates an alteration of relative risk for one unit of increase in the age category.

Similarly, 39 factors were evaluated as risk factors for VTE using pre‐, intra‐, and postoperative factors (Tables 4 and 7). The highest ORs were again for type of surgery, with esophagectomy (2.439) having the highest OR even when including postoperative factors, followed by sepsis (2.305), prolonged ventilation <48 hours (2.041), and readmission within 30 days (1.994).

**Table 4:** The risk for VTE associated with pre‐, intra‐, and postoperative factors (N: 382 124)

	VTE risk preOP + OP + Post OP			*P*
	β‐coefficient	Odds ratio	95% CI	
Age category	0.154	1.166	1.076‐1.264	<0.001
Intrahepatic bile duct cancer	0.353	1.424	0.912‐2.221	0.114
ASA (grade3‐5)	0.13	1.138	0.978‐1.325	0.097
No tumor	−0.3	0.741	0.584‐0.94	0.014
Hepatectomy performed for >1 segment apart from the lateral segment	0.674	1.962	1.587‐2.426	<0.001
Pancreaticoduodenectomy	0.483	1.621	1.33‐1.977	<0.001
Esophagectomy	0.892	2.439	1.987‐2.993	<0.001
Sex (male)	−0.454	0.635	0.567‐0.711	<0.001
Emergent surgery	0.478	1.613	1.266‐2.054	<0.001
Activities of daily living pre‐operation any assistance	0.012	1.012	0.848‐1.208	0.846
Pre‐operation ventilation	0.1	1.105	0.767‐1.592	0.492
Hypertension (any)	0.1	1.106	0.986‐1.239	0.085
Recent cerebrovascular disease	0.515	1.674	1.041‐2.689	0.031
Disseminated cancer	0.202	1.224	0.955‐1.569	0.111
Chronic steroid use	0.395	1.485	1.077‐2.048	0.014
Weight loss over 10%	0.285	1.33	1.098‐1.609	0.003
Bleeding disorder (any)	0.32	1.377	1.136‐1.669	0.001
Systemic sepsis	0.122	1.13	0.885‐1.443	0.276
BMI >25 kg/m^2^	0.473	1.604	1.403‐1.835	<0.001
BMI >30 kg/m^2^	0.239	1.27	0.96‐1.68	0.1
Platelet count >350 000/μL	0.25	1.284	1.084‐1.52	0.004
Serum albumin level <4 mg/dL	0.328	1.388	1.231‐1.566	<0.001
PT‐INR <0.9	0.371	1.449	1.078‐1.949	0.014
Operation time quartile over^a^	0.472	1.603	1.43‐1.797	<0.001
Reoperation	0.565	1.76	1.497‐2.069	<0.001
Overall SSI	0.37	1.447	1.236‐1.695	<0.001
Anastomotic leak	0.211	1.235	1.028‐1.484	0.024
Pancreatic fistula grade ABC	0.251	1.285	1.023‐1.616	0.033
Pneumonia	0.184	1.202	0.996‐1.451	0.011
Unplanned intubation	0.34	1.406	1.122‐1.761	0.004
Prolonged ventilation >48 h	0.714	2.041	1.616‐2.58	<0.001
Urinary tract infection	0.61	1.84	1.437‐2.357	<0.001
CNS occurrences any	0.226	1.254	0.998‐1.575	0.05
Cardiac occurrences any	0.65	1.915	1.485‐2.469	<0.001
Transfusion any	0.348	1.416	1.185‐1.692	<0.001
Septic shock	0.341	1.406	1.099‐1.8	0.008
Sepsis	0.835	2.305	1.81‐2.936	<0.001
SIRS	0.605	1.832	1.424‐2.356	<0.001
Readmission within 30 d	0.69	1.994	1.525‐2.608	<0.001

Abbreviations: ASA, American Society of Anesthesiologists Physical Status Classification; BMI, Body mass index; CNS, central nervous system; postOP, postoperation; PT‐INR, prothrombin time/international normalized ratio; SIRS, systemic inflammatory response syndrome; VTE, venous thromboembolism.

a Details of the time quartile over that shown in Table 7.

Similarly, 18 pre‐ and intra‐operative factors were evaluated for PE (Tables 5 and 7), and the results were similar to those for VTE.

**Table 5:** The risk for pulmonary embolism (PE) with pre‐ and intra‐operative factors (N = 382 124)

	PE risk preOP + OP			*P*
	β‐coefficient	Odds ratio	95% CI	
Age category^a^	0.223	1.25	1.112‐1.405	<0.001
Esophagectomy	1.173	3.231	2.313‐4.513	<0.001
ASA (grades 3‐5)	0.327	1.387	1.113‐1.729	0.004
Low anterior resection	0.398	1.488	1.14‐1.943	0.003
Hepatectomy performed for >1 segment apart from the lateral segment	0.965	2.626	1.957‐3.523	<0.001
Total gastrectomy	0.355	1.427	1.106‐1.84	0.006
Pancreaticoduodenectomy	0.905	2.473	1.867‐3.275	<0.001
Sex (male)	−0.286	0.752	0.634‐0.891	0.001
Emergent surgery	0.49	1.633	1.205‐2.214	0.002
Ascites	0.379	1.461	1.051‐2.032	0.024
Chronic steroid use	0.596	1.815	1.107‐2.976	0.018
Weight loss over 10%	0.512	1.669	1.263‐2.206	<0.001
Preop transfusions	0.554	1.739	1.24‐2.44	0.001
Systemic sepsis	0.712	2.037	1.412‐2.939	<0.001
Body mass index >25 kg/m^2^	0.76	2.138	1.784‐2.563	<0.001
Platelet count >350 000/μL	0.292	1.339	1.045‐1.717	0.021
Serum albumin level <4 mg/dL	0.372	1.45	1.211‐1.737	<0.001
Operation time quartile over^a^	0.651	1.918	1.621‐2.269	<0.001

Abbreviations: ASA, American Society of Anesthesiologists Physical Status Classification; OP, operation; preOP, preoperation.

Details of the time quartile over that shown in Table 7.

For PE, 28 pre‐, intra‐, and postoperative factors were selected (Tables 6 and 7). The highest OR was for any cardiac occurrence (2.926). Unplanned intubation (2.396) was the fourth highest. Chest events were recognized as high risk, which differed from risks for DVT.

**Table 6:** The risk for PE with pre‐, intra‐, and postoperative factors (N = 382 124)

	PE risk preOP + OP + postOP			*P*
	β‐coefficient	Odds ratio	95% CI	
Age category	0.122	1.129	1.003‐1.271	0.044
Esophagectomy	0.557	1.746	1.227‐2.483	0.002
ASA (grade3‐5)	0.043	1.044	0.829‐1.315	0.715
LAR	0.356	1.428	1.088‐1.875	0.01
Hepatectomy performed for >1 segment apart from the lateral segment	0.693	2	1.478‐2.705	<0.001
Total gastrectomy	0.129	1.137	0.876‐1.476	0.334
Pancreaticoduodenectomy	0.354	1.425	1.034‐1.964	0.03
Sex (male)	−0.44	0.644	0.542‐0.765	<0.001
Emergent surgery	0.169	1.184	0.866‐1.618	0.291
Ascites	0.204	1.226	0.88‐1.709	0.228
Chronic steroid use	0.476	1.609	0.978‐2.649	0.061
Weight loss over 10%	0.387	1.473	1.112‐1.951	0.007
preOP transfusions	0.331	1.392	0.987‐1.964	0.059
Systemic sepsis	0.207	1.23	0.846‐1.79	0.279
BMI >25 kg/m^2^	0.684	1.982	1.651‐2.38	<0.001
Platelet count >350 000/μL	0.325	1.384	1.077‐1.777	0.011
Serum albumin level <4 mg/dL	0.25	1.284	1.07‐1.541	0.007
Endoscopic surgery	−0.446	0.64	0.495‐0.827	0.001
Operation time quartile over^a^	0.533	1.705	1.433‐2.027	<0.001
Reoperation	0.56	1.75	1.371‐2.235	<0.001
Anastomotic leak	0.277	1.319	1.013‐1.717	0.04
Pancreatic fistula	0.478	1.614	1.159‐2.247	0.005
Pneumonia	0.499	1.647	1.253‐2.166	<0.001
Unplanned intubation	0.874	2.396	1.701‐3.374	<0.001
Prolonged ventilation >48 h	1.018	2.766	1.959‐3.906	<0.001
Urinary tract infection	0.51	1.665	1.133‐2.446	0.009
Cardiac adverse events (any)	1.074	2.926	2.138‐4.005	<0.001
Readmission within 30 d	1.037	2.82	1.975‐4.027	<0.001

Abbreviations: ASA, American Society of Anesthesiologists Physical Status Classification; OP, operation; postop, postoperation; preOP, preoperation; BMI, body mass index; CI, confidence interval; LAR, low anterior resection; PE pulmonary embolism.

a Details of the time quartile over that shown in Table 7.

**Table 7:** Duration of surgical procedures

	Mean (min)	Median (min)	Quartile over (min)
Gastrectomy	187	242	305
Total gastrectomy	210	267	338
Hepatectomy	393	367	477
Esophagectomy	481	469	573
Right hemicolectomy	203	188	245
Low anterior resection	275	254	335
Pancreaticoduodenectomy	470	455	547
Acute pan‐peritonitis surgery	129	115	160

### 3.5 Model performance

**Figure 1:**
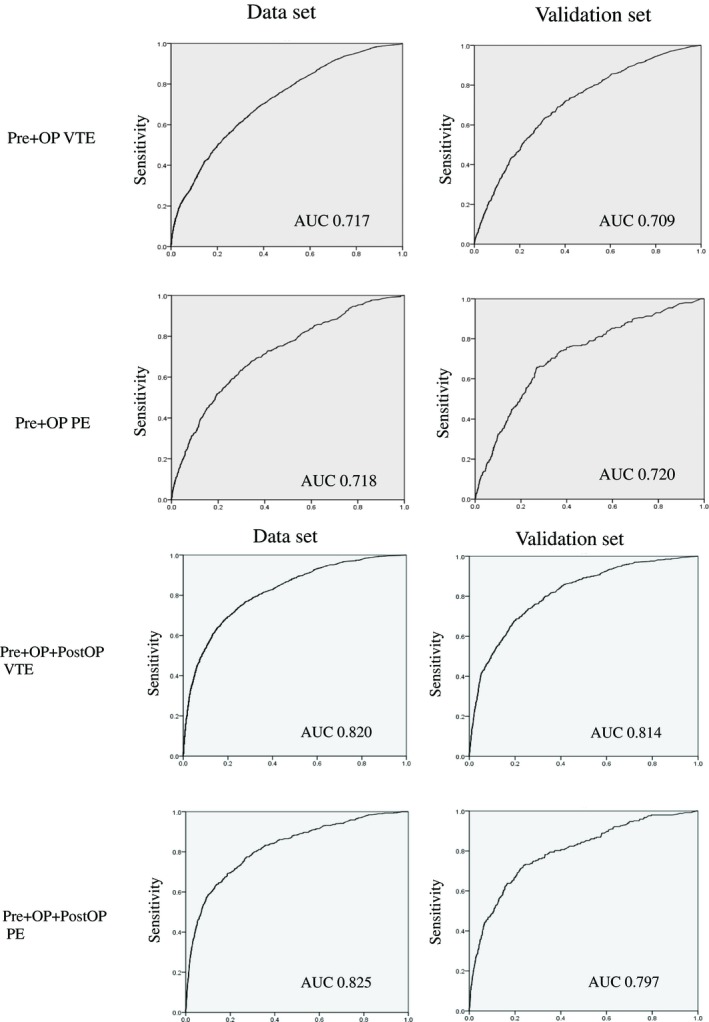
Receiver operating characteristic curve and area under the curve (AUC) of each data set. OP, operation; VTE, for venous thromboembolism; PE, pulmonary embolism

To evaluate model performance, the C‐index (a measure of model discrimination), which was the area under the ROC curve, was calculated for each data set in the models (Fig. 1). The C‐indices of the model for VTE and PE using pre‐ and intra‐operative factors were 0.717 (95% CI: 0.704‐0.731; *P *<* *0.007) and 0.718 (95% CI: 0.696‐0.739; *P *<* *0.011), respectively. The C‐indices of the model for VTE and PE using pre‐, intra‐, and postoperative factors were 0.820 (95% CI: 0.809‐0.832; *P *<* *0.006) and 0.825 (95% CI: 0.806‐0.843; *P *<* *0.009), respectively.

In the validation set models, the C‐indices of the model for VTE and PE using pre‐ and intra‐operative factors were 0.709 (95% CI: 0.687‐0.730; *P *<* *0.011) and 0.720 (95% CI: 0.687‐0.752; *P *<* *0.017), respectively. The C‐indices of the model for VTE and PE using pre‐, intra‐, and postoperative factors were 0.814 (95% CI: 0.795‐0.832; *P *<* *0.009) and 0.797 (95% CI: 0.767‐0.827; *P *<* *0.015), respectively (ROC depicted in Fig. 1). These data sets indicate the good discriminatory performance of the model.

### 3.6 Calibration of the model

**Figure 2:**
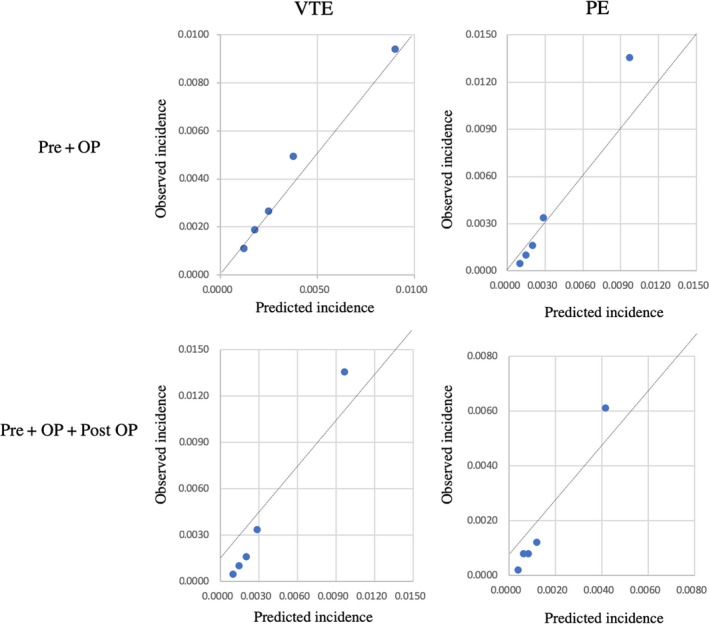
The calibration of the model for venous thromboembolism (VTE) or pulmonary embolism (PE). OP, operation

The predicted rate of VTE or PE using the risk model was highly consistent with the actual rate of onset in the validation data from the low‐risk group through to the high‐risk group (Fig. 2).

## 4 DISCUSSION

In this study, we used a database that included the largest number of registrations in Japan to investigate the frequency and risk factors for perioperative, symptomatic VTE and PE in clinical practice. The number of cases was 516 217, which was considerably larger than the number of cases in past reports.

In the eight surgical procedures assessed, the frequency of PE was 0.1%‐0.3%. The incidence was highest with the pan‐peritonitis operation and esophagectomy. The frequency of DVT was also 0.1%‐0.7%. The surgical procedures with the highest incidence of DVT were the pan‐peritonitis operation and esophagectomy. A previous study of patients in Japan reported that the incidence of perioperative PE during general surgery was 0.33%.^13^ The results of our study did not differ much from this past report. On the other hand, Rogers et al^14^ reported that the frequency of VTE was 0.93% after gastroenterological surgery in NSQIP data. This frequency is a little higher than the data from Japan and could be due to racial differences.

In this study, the frequency of PE was classified as low risk or high risk according to the 7th American College of Chest Physicians guidelines and Japan's guidelines. In these guidelines, cancer operations are classified as high risk. However, in the present study, among the surgical procedures, the risk was lower than that reported in the guidelines. However, the Japanese VTE risk score, the Wells score,^15^ and the Caprini score,^16^ which use Western VTE guidelines, do not include detail on the risk according to different types of surgical procedures. We found little relationship between our risk factor findings for VTE and those reported in the past. We found a seven‐fold difference in the frequency of PE based on the different surgical procedures. Moreover, differences in the risk factors for VTE and PE according to the type of surgical procedure were observed. These results show that the type of surgical procedure and the surgical site are important when considering the VTE risk factors.

In this study, we developed two different risk models to predict risk factors for VTE and PE. One used pre‐ and intra‐operative factors, while the other also included postoperative factors. The pre‐ and intra‐operative factors were sufficient to decide on peri‐operative prophylaxis. However, if risk factors were added after the operation, the risk of VTE and PE may change, although in past reports postoperative risk factors were only included in a few analyses of risk for VTE.

Venous thromboembolism and PE risk factors were different whether or not postoperative factors were added to the model. Intubation for more than 48 hours and re‐hospitalization were regarded as high‐risk factors for VTE, and chest events were recognized as risk factors for PE. These results show that it is necessary to reconsider the prophylaxis if complications occur after surgery, especially breathing and cardiac complications, which are important risk factors for PE.

This study has some limitations. We could not detect symptomatic versus asymptomatic VTE in the NCD data.

Prophylaxis has a great influence on the postoperative onset of VTE. However, the NCD has no information on what VTE prophylaxis was provided. Anticoagulant prophylaxis for VTE would provide important information about bleeding complications, but the NCD data does not include information about bleeding events associated with anticoagulant prophylaxis. The analysis of risk factors for VTE would be more accurate if the NCD database included such information and the kind of prophylaxis that was used.

In conclusion, the frequency of VTE and PE differed according to the type of surgical procedure (0.1%‐0.3% and 0.1%‐0.7%, respectively). Esophagectomy and pancreaticoduodenectomy were strong risk factors for VTE and PE. Also, the risk of VTE and PE also differed according to the type of surgical procedure. We recommend that these differences be reflected in the VTE risk score. By adding postoperative factors, VTE and PE risk factors were changed. It may be necessary to reconsider the prophylaxis if complications occur after surgery, especially breathing and cardiac complications, which are important postoperative risk factors for PE.

## DISCLOSURES

Funding: this study was supported by a clinical research grant from the Japan Surgical Society.

Conflicts of interest: authors declare no conflicts of interest for this article.

Author Contribution: Taishi Hata, Masataka Ikeda, Hiroaki Miyata, Masayoshi Nomura, Kouji Yamamoto and Yuichiro Doki make substantial contributions to conception and design, and/or acquisition of data, and/or analysis and interpretation of data. Taishi Hata, Masataka Ikeda, Hiroaki Miyata, Masayoshi Nomura, Kouji Yamamoto and Yuichiro Doki participate in drafting the article or revising it critically for important intellectual content. Taishi Hata, Masataka Ikeda, Hiroaki Miyata, Masayoshi Nomura, Mitsukazu Gotoh, Masato Sakon, Kouji Yamamoto, Go Wakabayashi, Yasuyuki Seto, Masaki Mori and Yuichiro Doki give final approval of the version to be published.
